# Lewontin’s Paradox Resolved? In Larger Populations, Stronger Selection Erases More Diversity

**DOI:** 10.1371/journal.pbio.1002113

**Published:** 2015-04-10

**Authors:** Roland G. Roberts

**Affiliations:** Public Library of Science, Cambridge, United Kingdom

## Abstract

A new study uses a massive comparative population genetics dataset to explore why the neutral genetic diversity of a species does not have a simple relationship to its population size.

Genetic variety is the spice of life—and it can also be the raw material upon which natural selection works its magic, shaping organisms to fit their circumstances. But there’s also the “dark matter” of genetic variation—neutral genetic diversity—that distinguishes individual genomes within a species from each other, but that has no impact on fitness. Despite its relative unimportance for evolution itself, neutral genetic diversity is of great interest to evolutionary biologists. It provides us with an unbiased picture of the history and population dynamics of a species, acting as a null hypothesis against which the effects of selection can be seen.

Under a simplistic neutral model (i.e., in the absence of selection), theoreticians might expect genetic diversity to scale in proportion to the total number of individuals in a population—a quantity known as the census population size. However, more than 40 years ago, the American evolutionary biologist Richard Lewontin noted that while population sizes of different species can vary across many orders of magnitude, the amount of neutral genetic diversity doesn’t, and indeed has no simple relationship to population size. How can this be?

This observation, which is often known as Lewontin’s paradox, has troubled theoretical and empirical biologists alike, and several potential explanations have been posited. In a new study just published in *PLOS Biology*, Russell Corbett-Detig, Daniel Hartl, and Timothy Sackton present persuasive empirical evidence for one mechanism that has had strong theoretical support but has hitherto been hard to put to the test—that natural selection is responsible for obliterating the expected relationship between diversity and population size.

When a genetic variant arises that confers a strong advantage on the host organism, natural selection ensures that its frequency will increase in the population, perhaps eventually becoming predominant. However, genes aren’t passed down the generations in isolation; instead, each is inherited along with sizeable chunks of neighbouring genomic regions. These genomic chunks are only broken up by the process of meiotic recombination, in which sexual organisms shuffle their two inherited genomes before passing them on to their offspring. Crucially, the consequence of this chunkiness is that selection, by acting on one functional variant, can also inadvertently change the frequency of tens or even hundreds of nearby neutral variants—a phenomenon known as hitchhiking. Thus, the effect of selection on a functional variant is to reduce the diversity of its genomic neighbourhood. A corresponding reduction in diversity is also seen around disadvantageous variants; a process known as background selection. Could these diversity-erasing effects of selection explain Lewontin’s paradox?

To address this, the authors took advantage of something that would have been inconceivable when Lewontin was writing: a massive accumulated wealth of genomic sequence diversity data from a wide range of organisms. They were able to select 40 plants and animals for which sufficient diversity data—plus a high-quality map of meiotic recombination rates—were available. Ranging from silkworm moth to watermelon and from baboon to orange (see Fig. 1), the authors believe that this is one of the largest comparative population genetics dataset ever assembled.

**Fig 1:**
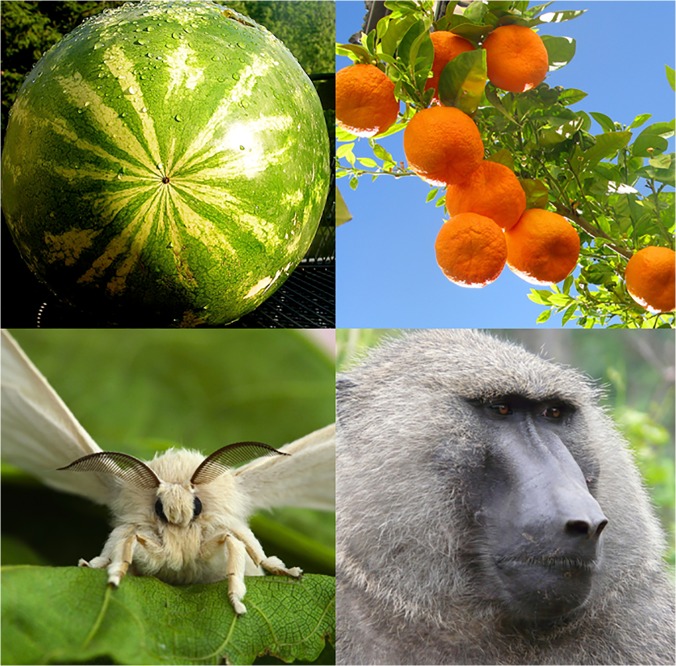
Organisms with large census population sizes—such as invertebrate animals (silkworm moth) and herbaceous plants (watermelon)—tend to experience a disproportionately more pronounced effect of selection on neutral genetic diversity than those with smaller population sizes—such as vertebrate animals (baboon) and woody plants (orange). Image credit: Flickr users Liz West (watermelon), Ronnie Macdonald (orange), Nikita (silkworm moth), and Mark Jordahl (olive baboon).

To assess the impact of natural selection on neutral genetic diversity, the authors compare regions of the genomes with high and low recombination rates (i.e., how big the genomic chunks are). The expectation is that where recombination rates are high (small chunks), selection will reduce diversity with the precision of an editor’s pencil; whereas, where recombination is rare (large chunks), selection will wipe broad swathes of diversity away like a chalkboard eraser. Comparing the two types of genomic regions should unmask the effects of selection.

Indeed, the authors find that in most species the relationship between recombination rate and diversity is incompatible with a purely neutral model but instead fits a mathematical model that incorporates the effects of hitchhiking and background selection. While the effects of selection on neutral diversity can be substantial, they vary between species in a way that matches our expectations of likely population size. Thus, selection tends to erase more diversity in invertebrates and herbaceous plants, which tend to be numerous, than in vertebrates and woody plants, which tend towards smaller population sizes. Assessing actual census population size is quite challenging, so the authors look at several well-established proxies for population size, geographical range, and body size. The measured impact of selection correlates positively with range and negatively with size, just as expected for a positive relationship with population size.

The authors conclude that in species with a large population size, selection plays a disproportionately high role in reducing neutral diversity, and a preliminary investigation suggests that while background selection (due to elimination of disadvantageous variants) is responsible for much of this reduction, it’s hitchhiking on the back of advantageous variants that largely accounts for the striking effects of population size.

As well as providing an empirical resolution to a longstanding paradox in population genetics and helping our understanding of the forces at work on the dark matter of genomic diversity, this realisation has important practical implications for those who depend on measures of neutral genetic diversity as a yardstick. Understanding the determinants of this parameter is central to many applications of population genetics, including the search for genes underlying common human diseases and for informing the conservation of threatened species; for them, this study presents the challenge as to how to incorporate the effects of hitchhiking and background selection into a useful neutral model.

